# Epidural Electrical Stimulation Within an Integrated Rehabilitation Pathway for Traumatic Cervical Spinal Cord Injury: Safety and 12-Month Functional Outcomes

**DOI:** 10.3390/jcm15187114

**Published:** 2026-09-14

**Authors:** Arzu Dinc Yavas, Aslihan Cevik Baran, Emir Eker, Seyma Sarioglu, Luay Serifoglu, Edip Gonullu, Ece Balkuv, Serif Onen, Gorkem Acar, Georgios Matis, Mustafa Kilic, İbrahim Asik, Akın Akakin, Dilek Akakin, Yakup Ozsezer, Halil Ulutabanca, Sevil Karagul, Shikhali Isgandarli

**Affiliations:** 1 Department of Physical Medicine and Rehabilitation, School of Medicine, Istanbul Aydın University, 34295 Istanbul, Türkiye; 2 Graduate Education Stem Cell and Tissue Engineering, Istinye University, 34396 Istanbul, Türkiye; 3 Department of Neurosurgery, School of Medicine, Istanbul Aydın University, 34295 Istanbul, Türkiye; aslihancevik@gmail.com; 4 Dr. Aslıhan Cevik’s Clinic, 34340 Istanbul, Türkiye; ekeremir8@hotmail.com (E.E.); seymasarioglu43@gmail.com (S.S.); 5 Department of Neurosurgery, Ümraniye Research and Education Hospital, 34766 Istanbul, Türkiye; luaybeyin@gmail.com; 6 Department of Algology, School of Medicine, Bakırçay University, 35665 İzmir, Türkiye; edipgonullu@gmail.com; 7 Department of Neurology, Üsküdar State Hospital, 34660 Istanbul, Türkiye; ecebalkuv@gmail.com; 8 Department of Physical Medicine and Rehabilitation, School of Medicine, Atlas University, 34408 Istanbul, Türkiye; serif.onen@atlas.edu.tr; 9 Faculty of Sports Science, Istanbul Gelisim University, 34310 Istanbul, Türkiye; 10 Interventional Pain Management, Spasticity and Neuromodulation Unit, Hygeia Hospital, 15123 Athens, Greece; gmatis@hygeia.gr; 11 Department of Neurosurgery, Medicana Zincirlikuyu Hospital, 34394 Istanbul, Türkiye; info@drmustafakilic.com.tr; 12 Division of Algology, Department of Anesthesiology and Reanimation, Faculty of Medicine, Ankara University, 06290 Ankara, Türkiye; iasik@ankara.edu.tr; 13 Department of Neurosurgery, Faculty of Medicine, Üsküdar University, 34768 Istanbul, Türkiye; drakinakakin@yahoo.com; 14 Department of Histology and Embryology, School of Medicine, Marmara University, 34854 Istanbul, Türkiye; dilekbangir@yahoo.com; 15 NEUROBIO Engineering, 34750 Istanbul, Türkiye; yakup@neurobioeng.com; 16 Department of Neurosurgery, Faculty of Medicine, Erciyes University, 38039 Kayseri, Türkiye; ulutabanca@erciyes.edu.tr; 17 Department of Physiotherapy and Rehabilitation, Faculty of Health Sciences, İstanbul Kent University, 34406 Istanbul, Türkiye; sevil.karagul@kent.edu.tr; 18 Department of Orthopedics and Traumatology, Central Hospital Kozyatağı, 34742 Istanbul, Türkiye; shixali448@gmail.com

**Keywords:** spinal cord injury, cervical spinal cord injury, epidural electrical stimulation, neuromodulation, neurorehabilitation, rehabilitation, functional recovery, therapeutic intervention

## Abstract

**Background/Objectives:** Epidural electrical stimulation (EES) is an investigational, off-label neuromodulation strategy used adjunctively with rehabilitation in chronic spinal cord injury (SCI), but safety and longitudinal functional data specific to traumatic cervical SCI remain limited. This is a descriptive safety-and-feasibility study, not an efficacy study; no efficacy or causal claim is made. **Methods:** In this single-centre observational cohort, 32 consecutive patients with traumatic cervical SCI underwent EES implantation and were included in baseline and safety analyses, while longitudinal analyses comprised the 26 participants with complete Spinal Cord Independence Measure (SCIM) III data at baseline and 3, 6, 9 and 12 months. Rehabilitation dose was quantified (approximately 1087 prescribed therapy hours over 12 months) and adverse events were classified using ISO 14155:2020 definitions with WHO–UMC causality grading. Prespecified outcomes were device- and procedure-related adverse events and total and domain-specific SCIM III scores; analyses were descriptive. **Results:** Patients were aged 33.8 ± 10.3 years, 28 (87.5%) were male, and baseline AIS grade was A in 31 and B in 1. Mean total SCIM III increased from 14.81 ± 8.92 at baseline to 21.77 ± 14.60, 25.12 ± 15.90, 28.23 ± 17.68 and 32.42 ± 19.45 at 3, 6, 9 and 12 months (all *p* < 0.001), with gains across self-care, respiration and sphincter management, and mobility; 10 of 32 patients (31.3%) improved by at least one AIS grade. Adverse events were infrequent: sterile hardware-site inflammation (2, 6.3%), explantation (1, 3.1%) and a first-ever seizure (1, 3.1%), without stimulation-related neurological deterioration. **Conclusions:** The cohort was highly selected (31/32 baseline AIS A; 87.5% male; injury-to-implant interval 10–339 months), no comparator group was available, stimulation programming was individualised rather than protocolised, and 6 of 32 patients lacked complete 12-month follow-up; these features limit reproducibility and generalisability and preclude any inference about effectiveness. EES with rehabilitation was well tolerated and accompanied by progressive functional gains, although the uncontrolled design precludes causal inference. The contribution of this report is therefore transparent safety, dose and programming data, together with a prespecified protocol framework intended to make a controlled, propensity-matched evaluation of EES in traumatic cervical SCI feasible and reproducible.

## 1. Introduction

The burden of spinal cord injury (SCI) extends beyond neurological deficit alone. Cervical injuries are particularly devastating because they frequently compromise upper-extremity function, in addition to deficits in trunk control, transfers, mobility, sphincter management, and overall independence in daily life [1,2]. The burden of SCI therefore includes chronic complications, reduced quality of life, and major social and economic consequences [3].

Neurological severity after SCI is commonly described using the American Spinal Injury Association (ASIA) Impairment Scale (AIS), but AIS does not fully capture day-to-day functional independence [1,4]. For this reason, functional outcome measures remain essential when evaluating recovery in SCI. The Spinal Cord Independence Measure (SCIM) was developed specifically for this population and assesses self-care, respiration and sphincter management, and mobility; it has been validated as a clinically meaningful outcome instrument in SCI cohorts [5,6,7]. In cervical SCI, even modest gains in SCIM domains may translate into clinically important improvements in transfers, self-care, and caregiver dependence.

Epidural electrical stimulation (EES) was originally introduced as a strategy for pain modulation [8], but over the past decade it has emerged as a promising, though still investigational, neuromodulatory approach for restoring function after SCI. Experimental and translational work has shown that stimulation can increase the excitability of spinal sensorimotor circuits, facilitate activation of residual descending pathways, and help re-engage locomotor networks below the lesion [9,10]. Landmark clinical studies further demonstrated that targeted epidural stimulation, particularly when combined with intensive rehabilitation, can support voluntary movement, standing, stepping, and overground walking even in selected individuals with chronic motor-complete SCI [10,11,12,13,14,15,16]. These reports are, however, small selected series or single cases; EES for motor and autonomic restoration is not an approved indication, and the devices used are regulatory-cleared for chronic pain and applied off-label in this setting.

At the same time, the EES literature remains heterogeneous. Studies differ with respect to electrode configuration, stimulation timing, rehabilitation intensity, neurological level, chronicity, and the specific outcomes reported [17]. These issues are particularly relevant in traumatic cervical SCI, where the loss of upper-extremity function compounds deficits in trunk control, transfer ability, bladder and bowel management, cough support, and other autonomic functions, so that clinically meaningful goals often extend well beyond stepping alone. Recent work on cervical-focused or non-invasive stimulation has reinforced the potential of stimulation-based approaches in tetraplegia, especially for arm and hand function [18], but data specific to traumatic cervical SCI remain limited.

Real-world safety and longitudinal functional data in traumatic cervical SCI specifically remain scarce, because most published EES experience derives from case reports or small, lower-limb-focused series. Documenting the safety profile and functional course of a comparatively large, single-centre cervical cohort is therefore a useful and necessary step before efficacy claims can be tested. We deliberately frame the present report as a descriptive safety-and-feasibility study rather than a prognostic analysis. We do report the observed correlation between 3- and 12-month SCIM III scores, but we interpret it cautiously: because these are two administrations of the same instrument in the same patients and both are strongly tied to baseline, a high correlation is expected as a matter of measurement stability and should not be read as a novel prognostic finding. Consistent with that framing, we also state at the outset what this report cannot do. It cannot establish efficacy; it cannot separate the contribution of stimulation from that of the intensive rehabilitation delivered alongside it or from residual spontaneous recovery; and, because programming was individualised rather than protocolised, the exposure as delivered cannot be reproduced by another centre. We therefore set out not only what was observed, but also the specific elements—quantified rehabilitation dose, standardised programming and reporting fields, blinded outcome assessment, and a matched comparator—that a subsequent controlled study would require in order to convert these observations into clinically actionable evidence.

We focused specifically on traumatic cervical SCI because this subgroup carries a particularly high functional burden and because even modest gains in self-care, transfer capacity, or sphincter management may meaningfully affect everyday life. The study was therefore designed as a descriptive safety-and-feasibility cohort study with two prespecified aims: (i) to characterise device- and procedure-related adverse events after EES implantation in all 32 implanted patients, and (ii) to describe the longitudinal course of total SCIM III scores over 12 months in the 26 participants with complete follow-up, within a combined, individualised neuromodulation-and-rehabilitation pathway. As secondary descriptive observations we report the correlation between 3- and 12-month SCIM III scores and whether AIS conversion co-occurred with larger SCIM III changes. Because programming and rehabilitation were individualised and no comparator group was available, we did not attempt causal or prognostic inference. The study also carries an explicitly methodological aim: (iii) to specify, on the basis of this experience, a transparent and reproducible protocol framework for the controlled evaluation of EES in traumatic cervical SCI, presented in Table 1.

## 2. Materials and Methods

### 2.1. Ethics Approval, Consent, and Regulatory Framework

The retrospective analysis of the clinical records was approved by the Non-Interventional Clinical Research Ethics Committee of İzmir Bakırçay University (Meeting decision number 2028; approval date 12 February 2025. To avoid the ambiguity noted at review, we confirm that “2028” is the sequential decision number issued by that committee and is not a calendar year, and that the approval date of 12 February 2025 precedes the preparation and submission of this manuscript). Because the injuries in this cohort date back several years and the analysis is retrospective, this approval covers the retrospective review and analysis of already-collected clinical data; the original implantations were performed as clinical care at the time of treatment, under consent for treatment. All procedures were conducted in accordance with the Declaration of Helsinki. Written informed consent for the surgical procedure and for the use of anonymised clinical data for scientific purposes was obtained from every participant, or from a legally authorised representative where the participant was unable to provide it in person. The Informed Consent Statement in the back matter, which previously read “None”, was an editorial error and has been corrected so that the two statements are consistent.

For completeness, and in response to the Editorial Office’s query, we confirm the sequence of events. The dates recorded in the submission system as the study period (1 February 2025 to 1 May 2025) denote the project period as a whole—protocol drafting, preparation and submission of the ethics application, retrospective record review, statistical analysis and manuscript preparation—and not a period of participant recruitment. No clinical record was accessed, and no data were extracted, anonymised or analysed for the purposes of this study, before the approval date of 12 February 2025; the interval between 1 and 12 February 2025 was occupied solely by preparation of the study protocol and submission of the ethics application. Ethics approval was therefore obtained before data collection for this study began. No prospective recruitment or enrolment of participants took place at any stage: every patient in this cohort had been treated years earlier as individualised clinical care, and the present study consists exclusively of the retrospective review of records that already existed at the date of approval.

Two patients required individual ethical consideration and are described here in full, so that the statements referring to them in the Results and Discussion have an explicit source in this section. Patient 27 was a 21-year-old man with a complete (AIS A) C2–C3 injury who was ventilator-independent but fully dependent for all activities of daily living, with a total SCIM III score of 0 at screening. Because the neurological level was higher than in the remainder of the cohort and the expected functional benefit was correspondingly uncertain, the indication was reviewed before implantation by a multidisciplinary board comprising neurosurgery, physical medicine and rehabilitation, neurology, algology, and specialist nursing. The board judged that the realistic therapeutic goals were autonomic and supportive—bowel and bladder facilitation, cough support, and spasticity control—rather than volitional motor recovery; that no alternative restorative option was available; and that the procedural risk was comparable to that in the remainder of the cohort. These goals, and the explicit possibility that no measurable SCIM III change would occur, were discussed with the patient and his family, and consent was given on that basis. The provenance of this record is stated explicitly in response to review: the board’s deliberation was documented prospectively and contemporaneously and was not reconstructed retrospectively for the purposes of this manuscript. The board met before implantation, and its assessment of the indication, the agreed autonomic and supportive therapeutic goals, the explicit statement that no measurable SCIM III change might occur, and the record of the discussion held with the patient and his family were entered in the medical record and in the pre-operative consent documentation on the date of that meeting, before the procedure was performed. The account given above is transcribed from those contemporaneous entries. The same applies to the board’s confirmation of eligibility for Patient 30, which was likewise recorded before implantation. He subsequently received the same stimulation-programming and rehabilitation pathway as the rest of the cohort, and his total SCIM III score remained 0 at every scheduled assessment; this outcome is reported unchanged rather than omitted. Patient 30 had a multilevel injury with documented cervical (C4) involvement together with additional thoracolumbar levels; the same board confirmed that the cervical involvement satisfied the inclusion criterion, and the patient was retained, with the multilevel pattern reported transparently in Section 2.2, examined in a sensitivity analysis in Section 3, and discussed in the Limitations.

The regulatory status of the intervention requires explicit statement. The implanted systems (Boston Scientific, Marlborough, MA, USA) hold regulatory clearance for chronic pain and were used off-label, with an explicit sensorimotor and autonomic restorative intent, at a time when no approved device existed for this indication in the treating jurisdiction. Off-label use was permitted under national provisions governing the use of an approved medical device outside its labelled indication where no approved alternative exists and the treating physician judges the expected benefit to outweigh the risk. Each implantation was therefore an individual clinical decision, documented in the medical record, taken after multidisciplinary review, and accompanied by a specific consent discussion covering: (i) the investigational nature of EES for motor and autonomic restoration and the absence of regulatory approval for that indication; (ii) the absence of any guarantee of functional benefit, including the possibility of no measurable change; (iii) surgical, hardware, and infection risks, including the possibility of explantation; (iv) the requirement for prolonged and intensive rehabilitation as a condition of implantation; and (v) the costs borne by the patient. No device manufacturer provided funding, devices, discounts, personnel, or in-kind support, and no author holds a consultancy, advisory, speaking, equity, or royalty relationship with the manufacturer of the implanted system (see Conflicts of Interest). We nevertheless accept the reviewer’s substantive point: individual off-label use, even where lawful, multidisciplinary, and fully consented, is a weaker governance framework than a prospective study with an approved protocol, prospective registration, an independent data and safety monitoring board, and predefined stopping rules. Our institution has consequently moved subsequent EES activity in this indication into a prospectively registered, ethics-approved protocol with independent safety monitoring, and we recommend that framework, rather than the one under which the present cohort was treated, for any further clinical use of EES for restorative indications.

### 2.2. Study Design and Patient Selection

**Figure 1:**
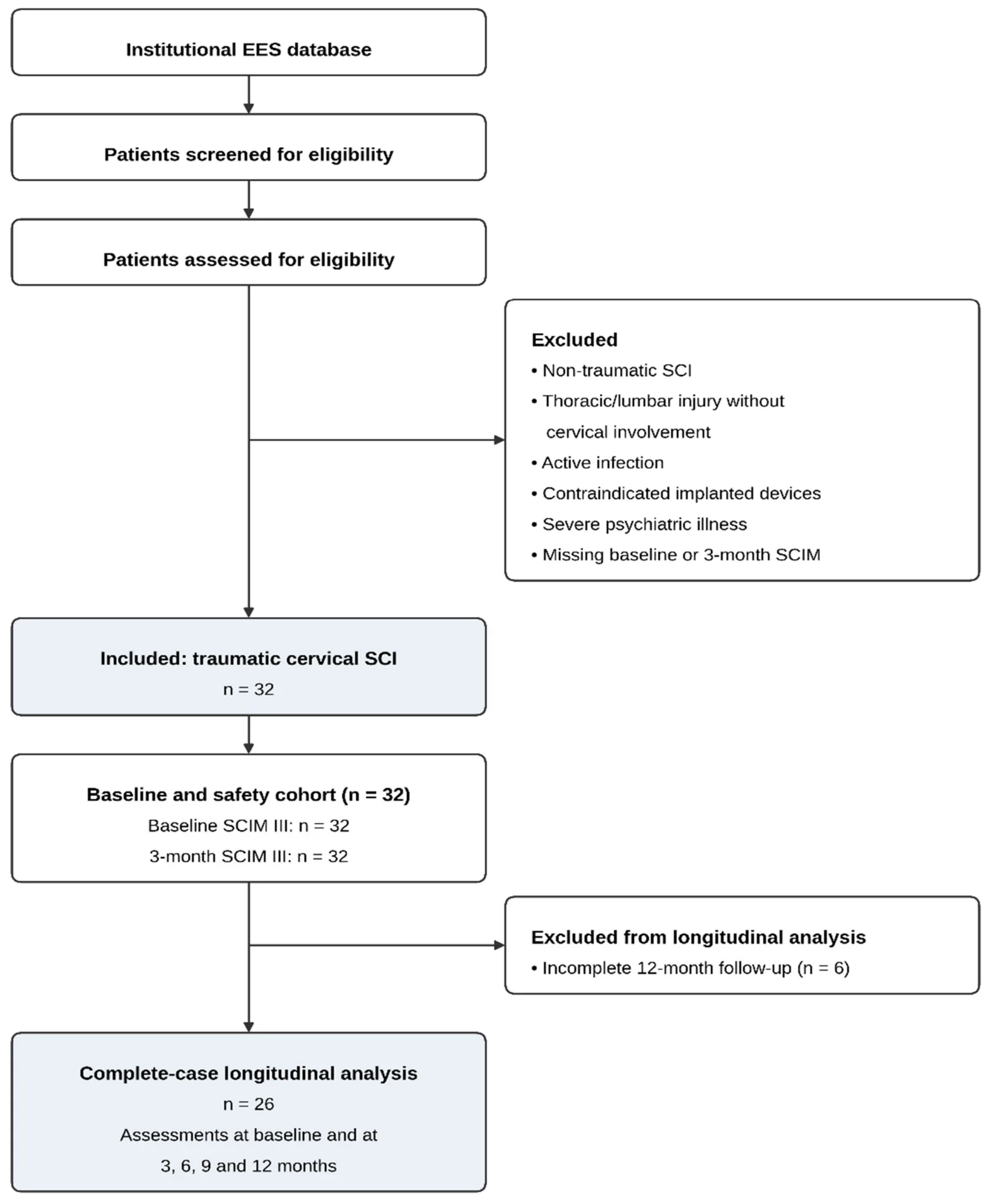
Study flow diagram. Patients were screened consecutively from the institutional EES database, and the number excluded is shown beside each exclusion reason (non-traumatic SCI, thoracic or lumbar injury without cervical involvement, active infection, contraindicated implanted electronic device, severe uncontrolled psychiatric comorbidity, neurological instability, missing baseline or 3-month SCIM III assessment, and cervical or combined cervical–thoracolumbar stimulation reported separately); 32 patients met the inclusion criteria for traumatic cervical SCI and formed the baseline and safety cohort; 26 of these patients completed SCIM III assessments at baseline and at 3, 6, 9, and 12 months and formed the complete-case longitudinal analysis cohort. EES, epidural electrical stimulation; SCI, spinal cord injury; SCIM, Spinal Cord Independence Measure.

This study was designed as a retrospective descriptive analysis of a prospectively followed clinical cohort of patients who underwent EES for chronic SCI, focusing specifically on traumatic cervical SCI. Thirty-two patients met the inclusion criteria and underwent EES implantation. The screening cascade is reported explicitly. Consecutive patients treated with EES were identified in the institutional database and assessed against the eligibility criteria in a fixed order: non-traumatic aetiology of the SCI; thoracic or lumbar injury without cervical involvement; active infection at screening; a contraindicated implanted electronic device; severe uncontrolled psychiatric comorbidity; neurological instability within the 3 months before implantation; absence of a baseline or 3-month SCIM III assessment; and treatment with cervical or combined cervical–thoracolumbar stimulation, which is reported separately. Thirty-two patients passed every step, underwent thoracolumbar EES implantation, and constitute the present cohort. Figure 1 shows the number of patients screened and the number excluded at each of these steps. Baseline characteristics and safety outcomes were analysed in the entire implanted cohort (*n* = 32), whereas longitudinal functional analyses were restricted to the 26 participants who completed SCIM III assessments at baseline and at 3, 6, 9, and 12 months (complete-case analysis; *n* = 26). The 6 patients excluded from the longitudinal analysis had incomplete 12-month follow-up; they contributed to the baseline and safety analyses only. Because a complete-case restriction of this kind can bias estimates upward if the patients without complete follow-up were also those doing less well, an intention-to-treat-style sensitivity analysis covering all 32 implanted patients is reported alongside the complete-case result: a conservative baseline-observation-carried-forward analysis in which the 6 patients without complete follow-up are assumed to have gained nothing. That analysis is specified in Section 2.9 and reported, together with the reasons for incomplete follow-up, in Section 3. The flow of patients through the study is summarised in Figure 1.

The implantations were performed as individualised clinical care, off-label, using regulatory-cleared spinal cord stimulation hardware; the present manuscript reports a retrospective analysis of the resulting records rather than a prospective interventional trial. Because the clinical use of EES in this cohort was delivered as individualised off-label therapy and not within a registered prospective trial, no trial-registration number applies. The study is reported in accordance with the STROBE (Strengthening the Reporting of Observational Studies in Epidemiology) guideline for cohort studies [22]. In all patients, the device was implanted with the explicit therapeutic goal of restoring sensorimotor and autonomic function after SCI, not for the management of chronic pain.

Patients were selected from an institutional database of individuals treated with EES. Inclusion criteria were traumatic cervical SCI (i.e., an injury involving one or more cervical segments), neurological stability for at least 3 months before implantation, availability of baseline and 3-month SCIM III assessments, and baseline AIS grade A–C at recruitment. The eligibility window was written as AIS grade A–C because that was the criterion applied prospectively in clinical practice; in the event, no patient with a baseline AIS grade of C was referred, screened, and implanted within the study period, so the realised cohort contains only AIS A (*n* = 31) and AIS B (*n* = 1) injuries. We report the criterion as it was applied rather than narrowing it retrospectively to match the realised sample, and we treat the resulting near-absence of baseline severity variation as a limitation. Patients with non-traumatic aetiologies, active infection, contraindicated implanted electronic devices, thoracic or lumbar injuries without cervical involvement, or severe uncontrolled psychiatric comorbidity were excluded. One patient (Patient 30) had a multilevel injury with documented cervical (C4) involvement together with additional thoracolumbar levels; because cervical involvement was present, this patient met the inclusion criterion and was retained, with the multilevel pattern described here in the text and considered in the limitations. The consequences of retaining this patient—for the segmental target of thoracolumbar stimulation, for the interpretation of SCIM III mobility items, and for the homogeneity of an already narrow baseline distribution—are set out once, in the fourth limitation in Section 4, and are not repeated here; exclusion of multilevel injuries is a prespecified eligibility criterion of the comparative study proposed in Table 2. Terminology has been standardised throughout the manuscript: “traumatic cervical SCI” is used wherever the study cohort or its target population is meant, and the unqualified term “cervical SCI” is retained only where a statement applies to cervical spinal cord injury irrespective of aetiology. Throughout the manuscript, patient identifiers (e.g., Patient 27, Patient 30) refer to the clinical numbering of the entire implanted cohort (Patients 1–32).

### 2.3. Clinical Data Collection

Demographic and clinical variables were extracted from the institutional data system, including age, sex, trauma mechanism, year of injury, implantation date, neurological level, baseline AIS grade, and target level of stimulation, for all 32 implanted patients. Longitudinal SCIM III assessments at baseline and at 3, 6, 9, and 12 months were available for the 26 participants who completed all scheduled follow-up visits and who comprised the functional analysis cohort.

### 2.4. Surgical Procedure

All patients underwent epidural spinal cord stimulator implantation under general anaesthesia using a standard sterile posterior midline approach. Following exposure of the relevant vertebral level, a limited laminectomy or hemilaminectomy was performed and a paddle-type surgical epidural lead (Boston Scientific, Marlborough, MA, USA) was inserted over the dorsal aspect of the spinal cord at the lumbosacral enlargement (T11–L1) under direct visualisation and fluoroscopic guidance. All leads in the present cohort were placed at this thoracolumbar target; within this fixed anatomical target, the final rostrocaudal lead position was fine-tuned according to each patient’s dominant functional goals.

The implantable pulse generator was tunnelled subcutaneously and positioned in a lumbar subcutaneous pocket. Perioperative antibiotic prophylaxis was administered according to institutional protocol.

### 2.5. Stimulation Programming

Stimulation programming was individualised for each patient and was not governed by a single fixed set of amplitude, frequency, or pulse-width values. Although the overall programmable range generally fell within 20–40 Hz for motor facilitation and 900–1200 Hz for inhibitory or spasticity-oriented paradigms, with pulse widths of 600–1000 μs and amplitudes of 1–25.5 mA, responses to any given stimulation combination varied substantially across patients, so the programming pattern was considered patient-specific rather than protocolised. The use of kilohertz-range frequencies for inhibitory or spasticity-oriented programming was guided by evidence that high-frequency spinal stimulation can suppress spinal excitability and reduce spasticity, although optimal parameters remain incompletely defined [25]. The wide amplitude range reflects the span of individually titrated settings across the whole cohort rather than values used in any single patient, and only a minority of patients were programmed at the upper end of this range.

We accept without reservation that the absence of a fixed, protocolised stimulation prescription is a principal limitation of this report. It reduces transparency, prevents independent replication of the exposure, and leaves open the possibility that programming decisions were influenced by the treating clinician’s expectation of benefit—a form of performance bias that a retrospective, unblinded design cannot exclude. Three steps have therefore been taken to make the exposure as reportable as it can retrospectively be made. First, the parameter space actually used is now reported in structured rather than narrative form: Table 1 lists, for each functional target, the electrode configuration, frequency band, pulse width, amplitude window, duty cycle, and the clinical endpoint used to titrate that program. Second, the titration rule that was in fact applied, although it was not written down prospectively, is stated explicitly: amplitude was increased from zero in 0.1–0.5 mA steps until the target muscle response became visible or palpable, then reduced to the lowest amplitude at which the intended functional endpoint (for example, knee extension sufficient for supported stance) was reproducibly achieved, and this threshold was re-checked at every monthly review. Third, we specify the programming protocol that we would now use, and that we recommend others use, in a prospective study: a fixed screening sequence of predefined contact configurations, standardised motor-threshold determination with surface electromyography, prespecified titration steps, and a locked parameter set held constant for a defined block before any change is permitted. Until such a protocol is applied, results obtained under individualised programming—including our own—should be read as descriptive observations rather than as a replicable intervention.

Programming was directed toward functionally important muscle groups and clinically meaningful autonomic targets. For standing and stepping, quadriceps activation was prioritised because knee extension is essential for stance stability, weight transfer, and gait initiation. Excessive hamstring activation, when it interfered with knee extension, was actively discouraged and, where necessary, treated with high-frequency inhibitory paradigms. Gluteal activation was pursued to improve pelvic and trunk stability, and ankle positioning and stepping-related lower-extremity activation were also used as functional targets.

Autonomic-oriented programming was incorporated in selected patients, especially for bladder and bowel management. In patients without preserved bladder sensation, palpable or visible suprapubic contractions were used as a practical clinical marker of detrusor activity during programming. For bowel programming, abdominal wall activation and facilitation of evacuation manoeuvres were similarly used when direct anorectal sensation was absent. Patients typically performed daily stimulation-assisted bladder or bowel attempts for approximately 15 min, followed by repeated attempts after temporary deactivation to assess carryover.

Upper-extremity programming followed functional priorities. Triceps activation was encouraged when elbow extension was needed for transfers and use of assistive devices; hand and finger flexor activation was emphasised when grasp was the priority. In selected patients, diaphragm and abdominal activation were considered clinically relevant for cough support and respiratory assistance.

In addition to function-specific motor, autonomic, and spasticity programs, individualised stimulation configurations were developed for different postures and daily activities, including dedicated programs for sitting, sleep, and cough facilitation. Low-frequency 2-Hz stimulation was also used in selected programs as a clearly perceptible rhythmic cue to alert the patient to stimulation onset or to signal transition to a specific functional task.

Depending on the intended clinical response, stimulation could be delivered through a single program or through multiple coordinated programs. In selected patients, four programs, designated A, B, C, and D, were activated concurrently or in programmed sequences to engage different electrode contact combinations and facilitate coordinated activation of multiple spinal networks and muscle groups. Additional temporal patterns, including Fast and Microburst modes, were incorporated when brief, rapid, or more selective activation was required. Fast stimulation sequences were used to support dynamic functional tasks requiring rapid transitions between muscle groups, whereas Microburst patterns consisted of short clustered stimulation trains intended to enhance selective neuromuscular recruitment.

All configurations were individualised according to the patient’s neurological status, posture, functional objective, muscle response, comfort, and tolerance. Programming was iterative, and stimulation settings were reassessed and modified during follow-up and rehabilitation as functional performance evolved.

### 2.6. Rehabilitation Program

All 32 patients participated in a structured, individualised rehabilitation program after EES implantation. The prescribed rehabilitation dose was quantified to enhance replicability. The supervised phase lasted 1 month and was scheduled 6 days per week, with Sunday reserved for rest, stretching, breathing exercises, positioning, and family education. Daily treatment consisted of four 50-min therapy sessions, totaling 20 h per week and approximately 80 h for the month. Additional tilt-table sessions were provided as needed for orthostatic intolerance. Robot-assisted gait training using the Lokomat^®^ Pro (Hocoma AG, Volketswil, Switzerland) and upper-extremity training using the Yeecon^®^ A6-2S Upper Extremity Rehabilitation Robot (Guangzhou Yeecon Medical Equipment Industrial Co., Ltd., Guangzhou, Guangdong, China) were each delivered twice weekly for 30 min, adding 4 h over the month. Thus, the total supervised dose was approximately 84 h. Following the supervised phase, patients transitioned to a 10-month home-based program. The home program was structured into five 40-min blocks performed daily, 7 days per week, totaling 23.3 h per week and approximately 1003 h over 10 months. The blocks included: (1) EES-assisted mat exercises; (2) trunk–hip and core control; (3) upper-extremity range-of-motion and task-specific training; (4) EES-assisted knee-extension and lower-limb facilitation; and (5) supported standing and balance activities. Caregivers were trained during the supervised phase to assist with the home program. Overall, the total prescribed rehabilitation dose over 12 months was 1087 h per patient. Adherence to the home program was monitored through weekly therapist telephone calls and patient-maintained logbooks. Estimated adherence was >75% in 20 of 26 patients with complete follow-up. Treatment fidelity was maintained by monthly in-person reassessment and program adjustment by the same senior rehabilitation therapist. Because this study reflects real-world clinical practice, adherence data were not captured with wearable sensors and are reported as estimates.

Twenty-four of the 32 patients had also continued a home-based maintenance exercise program during the chronic phase before enrolment. Importantly, the patients in this cohort were therefore not rehabilitation-naive; prolonged physical rehabilitation before implantation was virtually unavoidable, because long-term survival and daily management without rehabilitation are rarely feasible in this population. Accordingly, the post-implant phase represented a continuation and intensification of ongoing rehabilitation rather than the initiation of therapy in previously untreated patients. This intensive concurrent rehabilitation is a central reason why any functional change observed here cannot be attributed to stimulation alone.

### 2.7. Outcome Measures and Assessment Procedures

The prespecified primary outcomes were (i) device- and procedure-related safety events (hardware-site infection or inflammatory reaction, explantation, neurological deterioration, and any new adverse neurological event), analysed in the entire implanted cohort (*n* = 32), and (ii) longitudinal changes in total and domain-specific SCIM III scores, analysed in the 26 participants with complete follow-up. SCIM III assessments were performed at baseline and at 3, 6, 9, and 12 months after implantation. AIS grades were recorded at baseline, 3 months, and 12 months for the entire implanted cohort (*n* = 32). As secondary descriptive analyses, correlations between absolute 3- and 12-month SCIM III scores and between baseline and follow-up SCIM III scores were evaluated within the complete-case cohort (*n* = 26). These analyses were intended to describe longitudinal tracking of repeated measurements rather than to establish prognostic relationships.

Assessment conditions and assessor blinding are specified here in response to review. SCIM III assessments were performed by experienced physical medicine and rehabilitation clinicians belonging to the treating team, as part of routine clinical care. Because this is a retrospective analysis of a single-centre clinical service rather than a trial, the assessors were not blinded to the fact that the patient had been implanted, and independent blinded outcome assessment was not feasible; the risk of observer and assessment bias is therefore real, is not mitigated by the design, and is stated as a limitation. Two procedural safeguards were nevertheless in place and are documented in the clinical record: SCIM III was scored from directly observed task performance using the standard published scoring rules rather than from patient self-report, and the clinician who scored the instrument was not the clinician responsible for stimulation programming, so scoring was not performed by the person whose programming decisions were being evaluated. The stimulation condition at the time of assessment is also specified. SCIM III is an instrument of everyday functional independence, and all assessments were accordingly performed in the patient’s usual daily-use condition, that is, with the device active and running the patient’s habitual daily programs. No stimulation-off assessments were performed. The reported scores therefore describe function with stimulation in use, and cannot be decomposed into a durable functional change and an immediate stimulation-dependent increment. A stimulation-on versus stimulation-off assessment performed by an assessor blinded to condition is required to make that separation, and it is incorporated into the protocol framework proposed in Table 1.

### 2.8. Adverse Event Definitions, Causality Assessment, and Surveillance Windows

Adverse events were defined and classified retrospectively using the definitions of ISO 14155:2020 [20]. An adverse event was any untoward medical occurrence in a participant after implantation, irrespective of its relationship to the device or the procedure. A serious adverse event was an event that led to death, to serious deterioration in health requiring inpatient hospitalisation or prolongation of an existing hospitalisation, to medical or surgical intervention to prevent permanent impairment of a body structure or function, or to life-threatening illness or injury. The terms device deficiency, adverse device effect, and serious adverse device effect were applied as defined in the same standard. Causality between each event and the device or the implantation procedure was assessed independently by two clinicians—a neurosurgeon and a rehabilitation physician—using the WHO–UMC standardised case causality categories (certain, probable, possible, unlikely, conditional/unclassified, unassessable), with disagreement resolved by consensus [21]. Severity was graded as mild, moderate, or severe according to the level of intervention required. The identity and the independence of the adjudicators are specified here in response to review. The two assessors were a consultant neurosurgeon and a consultant physical medicine and rehabilitation physician of the treating institution. Each recorded a causality grade independently, on a separate proforma and without sight of the other’s assessment, before the two gradings were compared and any disagreement was resolved by consensus. Neither assessor adjudicated an event in a patient for whom he or she had made the stimulation-programming decisions, so no clinician graded the causality of an event arising from programming that he or she had prescribed. We state plainly, however, the limit of that arrangement. Both assessors belong to the same institution and to the wider treating service, and the adjudication was therefore internal: it was independent of the operating surgeon and of the programming clinician, but not of the centre, and it cannot be regarded as free of institutional interest in the results. Fully external adjudication, by clinicians with no involvement in the care of this cohort, was not available for a retrospective analysis of this kind; it is specified as a requirement of the prospective study proposed in Table 2 and of the reporting framework in Table 1.

Two surveillance windows are distinguished throughout, because conflating them was a source of the inconsistency identified at review. Functional outcomes were collected within a fixed 12-month window (baseline and 3, 6, 9, and 12 months). Safety surveillance was not restricted to that window: device- and procedure-related events were captured continuously through routine clinical follow-up for as long as the patient remained under institutional care, and in every patient for whom surveillance extended beyond the 12-month functional window. Every event is reported with its timing relative to implantation, and events occurring after month 12 are identified as such. Applying these definitions to the present cohort, the two persistent sterile hardware-site inflammatory reactions were classified as adverse device effects of probable causality, graded moderate in one patient (managed conservatively) and severe in the other (requiring explantation); the explantation, which occurred approximately 18 months after implantation and therefore outside the functional-assessment window but inside the safety-surveillance window, was classified as a serious adverse device effect; and the first-ever epileptic seizure was classified as a serious adverse event of possible causality. It was retained in the “possible” rather than the “unlikely” category because no alternative cause was identified, even though no established biological mechanism links thoracolumbar epidural stimulation to cortical epileptogenesis. Because the reviewer asks for that grading to be justified against the criteria themselves rather than asserted, the reasoning is set out in full. Under the WHO–UMC system, “possible” denotes an event with a reasonable temporal relationship to the exposure, which could also be explained by concurrent disease or by other drugs or devices, and for which information on withdrawal of the exposure is lacking or unclear. “Unlikely” requires more than the mere absence of a demonstrated mechanism: it requires a temporal relationship that renders a causal connection improbable and, in addition, other drugs, devices, chemicals, or underlying disease that provide a plausible alternative explanation. Both limbs of that definition must hold, and in this case neither did. The temporal relationship did not make causality improbable, because the event occurred while the implanted system was in continuous daily use rather than before implantation or after explantation. No plausible alternative explanation was established either: the patient had no personal or family history of epilepsy and no antecedent febrile or symptomatic seizure, and the diagnostic work-up carried out at the time of the event did not identify a structural, metabolic, toxic, infectious, or drug- or alcohol-related precipitant, although an interictal EEG abnormality was demonstrated. To assign “unlikely” in that situation would have asserted a confidence in non-relatedness that the record does not support. The categories above “possible” were equally inapplicable: “probable” additionally requires a clinically reasonable response to withdrawal of the exposure, and neither dechallenge nor rechallenge was performed, because stimulation remained clinically indicated and its withdrawal was not judged necessary; and, as noted, no established biological mechanism links thoracolumbar epidural stimulation to cortical epileptogenesis, so there is no mechanistic argument for a higher grade. “Possible” is therefore the category that the available information supports, and it was assigned as the conservative option, erring towards over- rather than under-reporting a potential device-related risk. No device deficiency, lead migration, lead fracture, cerebrospinal fluid leak, or new neurological deficit attributable to stimulation was recorded in either window.

### 2.9. Statistical Analysis

Continuous variables were summarised as mean ± standard deviation (SD) or median (interquartile range [IQR]), as appropriate, and categorical variables as frequencies and percentages. Baseline demographic characteristics and safety outcomes were analysed for the entire implanted cohort (*n* = 32). Longitudinal analyses of total and domain-specific SCIM III scores were restricted to the 26 participants with complete follow-up (complete-case analysis). Changes from baseline to each follow-up time point were assessed using the Wilcoxon signed-rank test. Because repeated comparisons were performed across five time points, these analyses were considered descriptive and interpreted with Holm correction where appropriate. Correlations between absolute SCIM III scores at different follow-up time points were evaluated using Pearson correlation coefficients with corresponding 95% confidence intervals, with Spearman coefficients reported as a sensitivity check. No multivariable regression modelling was performed because of the limited sample size and the highly homogeneous baseline AIS distribution. Statistical significance was defined as a two-sided *p* value < 0.05. All statistical analyses were performed using IBM SPSS Statistics version 29.0 (IBM Corp., Armonk, NY, USA). We accept that repeated cross-sectional comparisons discard the repeated-measures structure of the data and force a complete-case restriction, and that a linear mixed-effects model would be the more efficient use of a longitudinal design; such a model is accordingly specified as the primary analysis of the prospective comparative study proposed in Table 2 [26]. In the present retrospective dataset, however, the missing data are monotone and confined to 6 of 32 patients, and a model-based estimate would rest on a missing-at-random assumption that these data cannot test. We therefore report an assumption-light alternative that covers the entire implanted cohort. The complete-case Wilcoxon comparisons are retained as the descriptive summary of the 26 participants with complete follow-up, and are accompanied by a conservative baseline-observation-carried-forward analysis of all 32 implanted patients, in which every missing post-baseline observation is replaced by that patient’s own baseline value. The 6 patients without complete follow-up are thereby assumed to have gained nothing, so the resulting estimate is a lower bound on the change across the implanted cohort rather than an estimate of the change in the completers. The 10-point threshold used to define a substantial response is not an arbitrary choice and is now referenced. Scivoletto et al. derived distribution-based estimates for the SCIM and reported a minimal clinically important difference of approximately 4 points and a minimal detectable change of approximately 8 points for the total score, identifying a change of 10 points as corresponding to a significant and substantial improvement [19]; both thresholds are applied in Section 3. Exact Holm-adjusted *p* values are reported to three decimal places, and the notation *p* < 0.001 is used only where the adjusted value is genuinely smaller than 0.001. All tests were two-sided. The software used for each analysis is specified here in response to review. All analyses of the present cohort—descriptive statistics, Wilcoxon signed-rank comparisons with Holm correction, Pearson and Spearman correlations, and the baseline-observation-carried-forward sensitivity analysis—were performed in IBM SPSS Statistics version 29.0 (IBM Corp., Armonk, NY, USA). No propensity-score matching was carried out in the present study, because no comparator group could be assembled; the matching specification in Table 2 belongs to the prospective comparative study and has not been executed. For that study the matching will be performed in R version 4.4.1 or later (R Foundation for Statistical Computing, Vienna, Austria), using the MatchIt package (version 4.7.2) for nearest-neighbour matching on the logit of the propensity score with a calliper of 0.2 standard deviations and exact matching on baseline AIS grade [24], the cobalt package for standardised mean differences before and after matching, and the lme4 and lmerTest packages for the linear mixed-effects model. The propensity-score model, the matching call, and the full analysis script will be deposited with the study protocol so that the specification is independently reproducible.

## 3. Results

Thirty-two patients met the inclusion criteria and underwent EES implantation. Baseline characteristics and safety outcomes were analysed in the entire implanted cohort (*n* = 32). Twenty-six participants completed all scheduled SCIM III assessments through 12 months and comprised the longitudinal functional analysis cohort (*n* = 26); the remaining 6 patients had incomplete 12-month follow-up and were therefore excluded from the longitudinal analyses only. The patient flow is shown in Figure 1.

The mean age of the implanted cohort was 33.8 ± 10.3 years (median 30 years; range 21–53), and 28 patients (87.5%) were male. Baseline AIS grade was A in 31 patients and B in 1 patient. Baseline demographic and clinical characteristics of the entire implanted cohort are summarised in Table 3. The cohort included Patient 27, a 21-year-old with a complete C2–C3 injury, whose indication, multidisciplinary review, consent process, and management are described in full in Section 2.1 and whose outcome is considered in the Discussion. Motor vehicle traffic injury was the most common trauma mechanism, followed by shallow-water diving and high-energy falls.

At implantation, the cohort represented a predominantly chronic population. The mean injury-to-implant interval was 64.3 ± 66.7 months, with a median of 57 months and a range of 10–339 months (Table 1).

Safety observations refer to all 32 implanted patients and should not be extrapolated to the broader institutional EES cohort. Safety and complication data were available for all 32 implanted patients, because device- and procedure-related events were captured through routine clinical care. EES implantation was generally well tolerated. Each event below is reported with the ISO 14155:2020 category, the WHO–UMC causality grade, and the time of occurrence relative to implantation, as defined in Section 2.8. Of the 32 implanted patients, 2 (6.3%) developed persistent sterile inflammatory reactions localised to the hardware site; of these, 1 (3.1%) required device explantation for persistent, unresolved inflammation, while conservative management was sufficient in the other. One patient (3.1%) experienced a first-ever epileptic seizure within the 12-month follow-up window, with an EEG abnormality and no prior history of epilepsy, and was managed with antiepileptic treatment. Because a causal relationship to stimulation cannot be established or excluded from a single retrospective case, this event is reported conservatively as a serious adverse event of uncertain relationship to the device. Device- and procedure-related safety was monitored through routine clinical follow-up that continued beyond the 12-month functional assessment window; the explantation described above occurred during this extended surveillance, approximately 18 months after implantation. No stimulation-related neurological deterioration or permanent device-related neurological complication was observed. The overall explantation rate was 3.1% (1/32).

Among the 26 participants with complete follow-up, mean total SCIM III increased from 14.81 ± 8.92 at baseline to 21.77 ± 14.60 at 3 months, 25.12 ± 15.90 at 6 months, 28.23 ± 17.68 at 9 months, and 32.42 ± 19.45 at 12 months (Table 2). The increases from baseline to each follow-up assessment were statistically significant (Wilcoxon signed-rank test with Holm correction; every Holm-adjusted *p* value was below 0.001, and the basis for reporting these values against that threshold rather than as point estimates is given in the footnote to Table 4).

**Figure 2:**
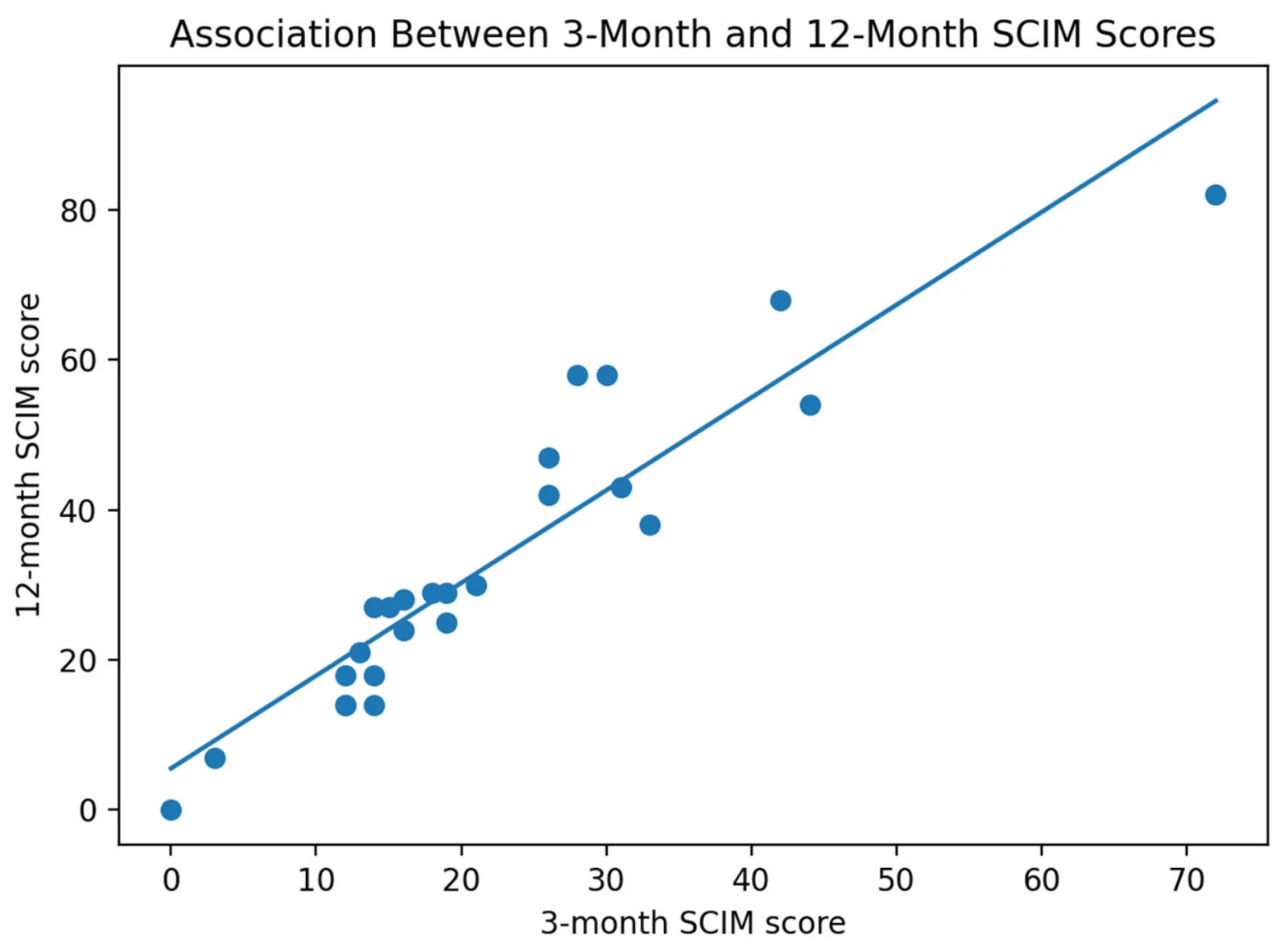
Association between absolute 3-month and 12-month total SCIM III scores in participants with complete 12-month follow-up (*n* = 26). Each point represents one participant; the line indicates the least-squares fit (Pearson r = 0.93; 95% CI 0.86 to 0.97). SCIM, Spinal Cord Independence Measure.

In the 26 participants with complete follow-up, absolute 3-month and 12-month SCIM III scores were strongly correlated (Pearson r = 0.93; 95% CI 0.86 to 0.97; Spearman ρ = 0.95; Figure 2). However, baseline SCIM III was itself already correlated with both the 3-month (r = 0.81) and the 12-month (r = 0.80) score. The 3-to-12-month relationship therefore largely reflects stable between-patient differences that were fixed at baseline—that is, tracking of a repeated instrument—rather than new information contributed by the 3-month assessment. We report this correlation only as a descriptive property of the data and draw no prognostic conclusion from it. A covariate-adjusted regression model was deliberately not fitted, because at this sample size such a model would be non-identifiable and its coefficients uninterpretable; formal, penalised, and validated prognostic modelling is deferred to an adequately powered, prospectively designed study.

**Figure 3:**
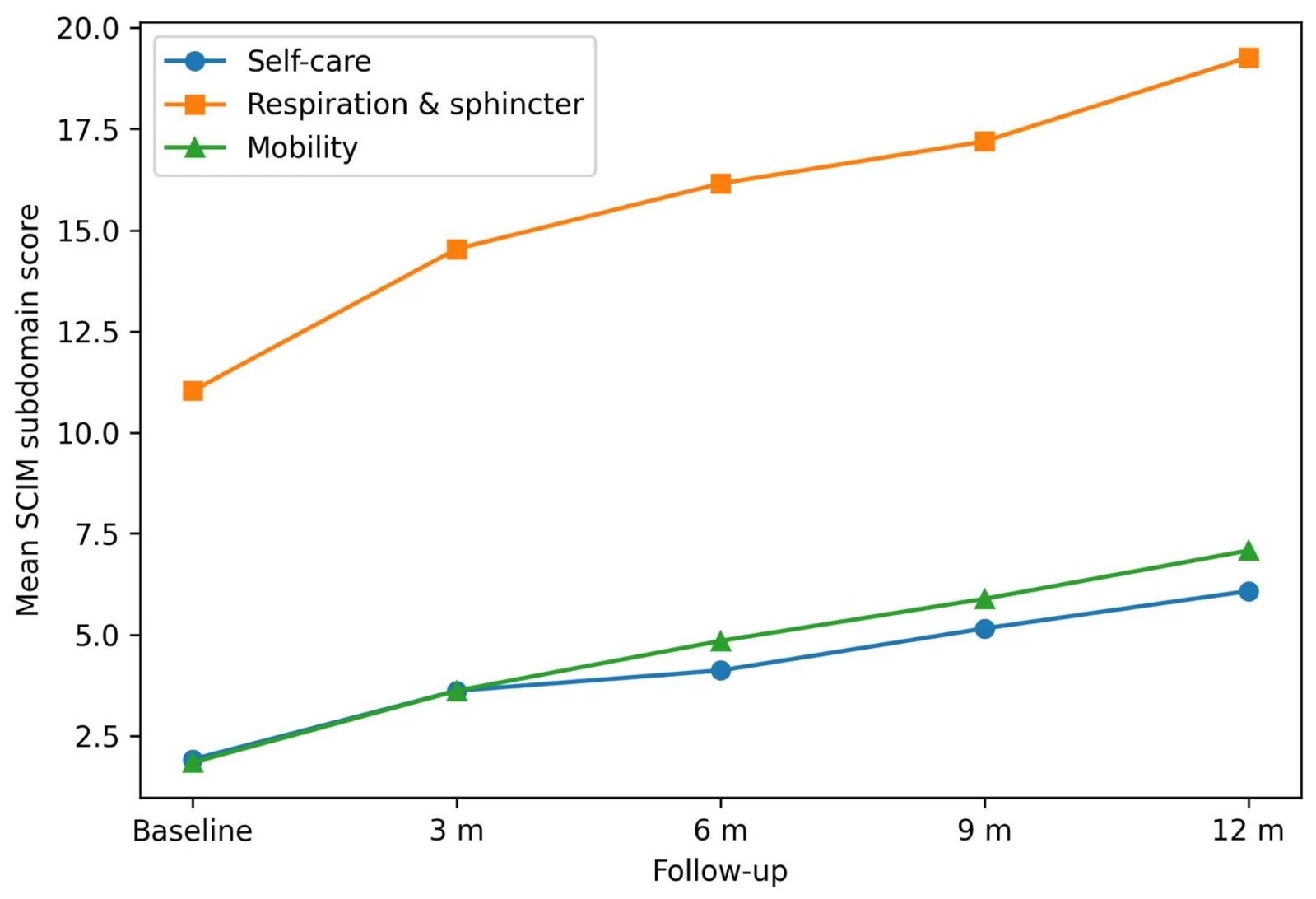
Mean SCIM III subdomain scores at baseline and at 3, 6, 9, and 12 months in participants with complete 12-month follow-up (*n* = 26). SCIM, Spinal Cord Independence Measure.

Among the 26 participants with complete longitudinal SCIM III domain data, functional improvement was observed across all domains (Table 5). Mean self-care scores increased from 1.92 ± 2.74 at baseline to 6.08 ± 5.31 at 12 months. Respiration and sphincter management improved from 11.04 ± 4.85 to 19.27 ± 9.20, while mobility increased from 1.85 ± 2.39 to 7.08 ± 6.04. These findings indicate that longitudinal functional improvement was not confined to mobility but also involved activities of daily living and autonomic function (Figure 3).

At the individual level, the mean change in total SCIM III from baseline to 12 months was +17.62 ± 13.10 points (95% CI 12.33 to 22.91; median +13.5, IQR 7.5–21.0), ranging from no change to an increase of 52 points. Eighteen of the 26 participants (69.2%) improved by at least 10 points, whereas 8 (30.8%) improved by less than 10 points (Table 6). The 10-point threshold is not arbitrary: it corresponds to the change identified by Scivoletto et al. as a significant and substantial improvement in total SCIM, against a minimal clinically important difference of approximately 4 points and a minimal detectable change of approximately 8 points [19]. Because the 10-point criterion is more demanding than either of those thresholds, the 18 participants (69.2%) who improved by at least 10 points necessarily exceeded both the 4-point minimal clinically important difference and the 8-point minimal detectable change; the mean change of +17.62 points (95% CI 12.33 to 22.91) and the median change of +13.5 points likewise exceed both. Because these thresholds are distribution-based and were derived in a predominantly subacute, mixed-severity SCI population rather than in chronic motor-complete cervical injury, they are used here to aid interpretation and not as evidence of treatment effect.

Twelve-month AIS grade was available for all 32 implanted patients (AIS A, 22; B, 5; C, 4; D, 1). Ten of these 32 patients (31.3%) converted to a better AIS grade by 12 months. Within the 26 participants who also had 12-month SCIM III data, mean 12-month total SCIM III increased monotonically across 12-month AIS grades, and participants who converted to a better AIS grade had a mean 12-month SCIM III approximately double that of participants without AIS conversion. Given the small numbers and the predominance of baseline AIS A, these AIS-based comparisons are descriptive only and were not formally tested. Because one patient with a baseline AIS B injury showed an exceptionally large improvement (SCIM III 30 to 82; conversion to AIS D), the converter–non-converter contrast and the upper end of the 3-to-12-month correlation are influenced by a small number of high-responding patients and should be read with corresponding caution.

Outcomes disaggregated by sex were considered and are not reported, and the reason is stated here explicitly rather than left implicit. Four of the 32 implanted patients (12.5%) were women, and the number of women within the 26-participant complete-case cohort is smaller still. A group of that size cannot support a meaningful summary statistic: a mean would be determined by individual variation rather than by any property of the group, a confidence interval would span much of the observable range of the instrument, and a comparison with the male group would have negligible power, so that a non-significant result would be uninformative and a significant one unreliable. Reporting individual trajectories for so few patients would in addition risk re-identification within a single-centre cohort in which sex, age, neurological level, and trauma mechanism are already reported. We therefore state what is accurate—that these data can support no statement whatever about the response to EES in women, neither that it resembles nor that it differs from the response in men—rather than presenting a descriptive contrast that would invite an inference the sample cannot bear. Sex-disaggregated reporting, with a prespecified minimum enrolment of women, is accordingly a requirement of the prospective study specified in Table 2 and of the reporting framework in Table 1, and the near-absence of women from this cohort is restated as the second limitation in Section 4.

**Figure 4:**
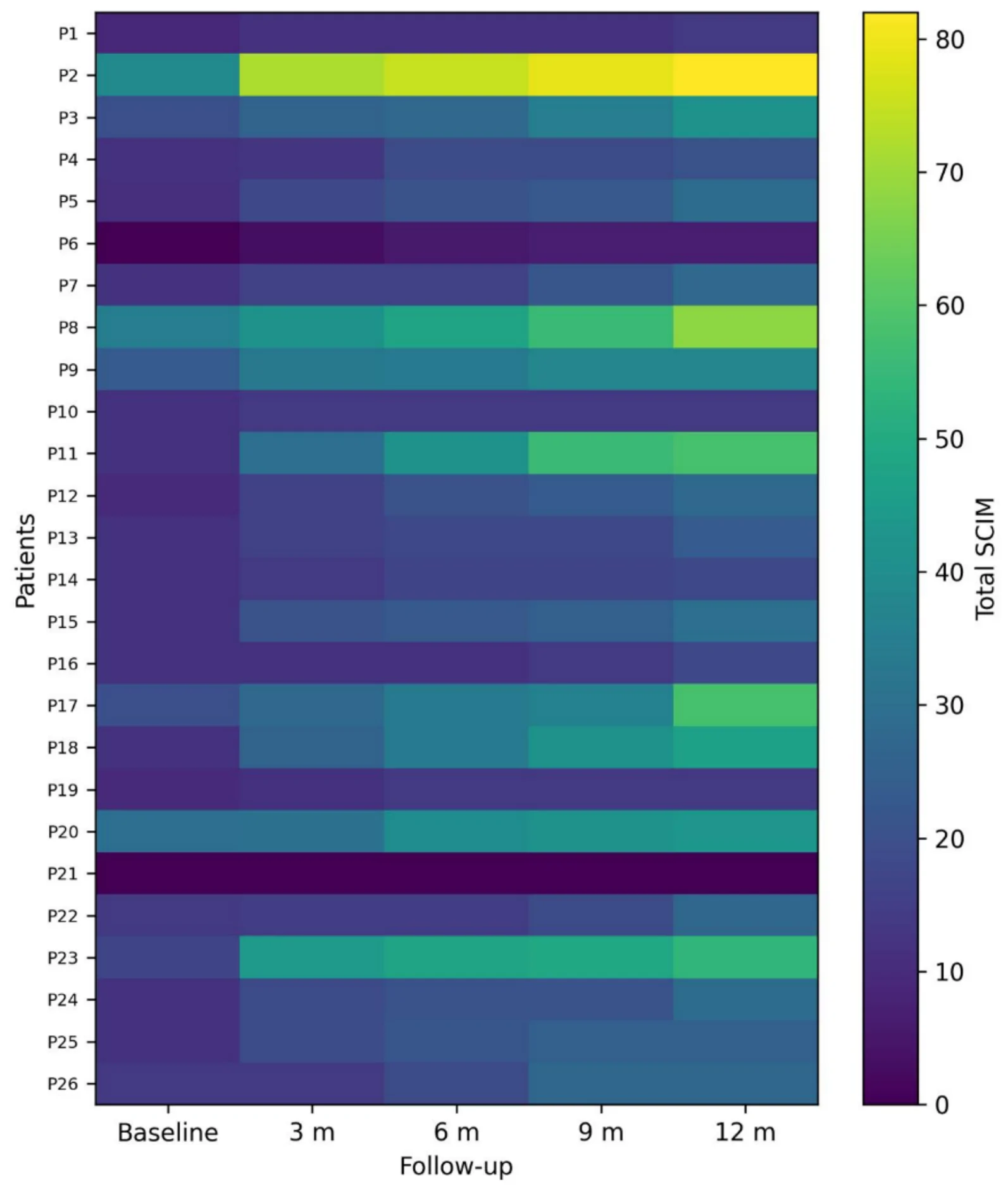
Heat map of individual total SCIM III trajectories in participants with complete longitudinal follow-up (*n* = 26). Each row represents one participant and each column a scheduled assessment (baseline, 3, 6, 9, and 12 months). The colour scale represents the total SCIM III score. A perceptually uniform, colour-vision-deficiency-safe sequential colour map is used, and the numeric total SCIM III value is printed inside every cell, so that the figure can be read without reliance on colour discrimination. Participants are numbered sequentially (P1–P26) within the complete-case cohort; these identifiers are independent of the clinical patient numbers used elsewhere in the text (Patients 1–32). SCIM, Spinal Cord Independence Measure.

Individual patient trajectories are illustrated in the heat map (Figure 4), in which each row represents one participant of the complete-case cohort and each column represents a scheduled assessment (baseline, 3, 6, 9, and 12 months). The colour intensity reflects the total SCIM III score (0–100), with darker colours indicating lower and brighter colours indicating greater functional independence. The heat map demonstrates substantial inter-individual variability in longitudinal functional trajectories despite an overall trend toward progressive improvement.

These results are descriptive and hypothesis-generating. The cohort characteristics that constrain their interpretation—the near-uniform baseline AIS distribution, the individualised stimulation programming and rehabilitation, and the absence of any comparator group—are enumerated once, in the Limitations in Section 4, and are not restated here. This study was not designed to demonstrate efficacy or to establish a prognostic or predictive model, and it should not be read as doing so.

Because restricting the longitudinal analysis to 26 of the 32 implanted patients raises the possibility of attrition and selection bias, the 6 patients without complete 12-month SCIM III data are accounted for here rather than merely counted. All 6 were implanted, were followed clinically, and contributed complete baseline and safety data; their exclusion from the longitudinal analysis reflects missing scheduled SCIM III assessments rather than withdrawal from treatment, and none was withdrawn because of a device- or procedure-related adverse event. Because the number of non-completers is small and their post-baseline trajectories are unknown, we do not assert that attrition was non-differential, and no formal completer versus non-completer comparison of outcome is possible. Instead, the effect of the missing data on the reported result is bounded directly in the following paragraph.

To ensure that the complete-case figure is not read in isolation, the 12-month change was recalculated across all 32 implanted patients using the conservative baseline-observation-carried-forward approach specified in Section 2.9, in which each of the 6 patients without complete follow-up is assigned no change from baseline. On this basis the mean change in total SCIM III from baseline to 12 months was +14.32 ± 13.68 points (95% CI 9.38 to 19.25), compared with +17.62 ± 13.10 points (95% CI 12.33 to 22.91) in the 26 participants with complete follow-up. The two estimates therefore bracket the cohort result: +17.62 points describes the participants who completed the assessment schedule and is the most favourable of the available estimates, whereas +14.32 points is a lower bound for the implanted cohort under the assumption that every patient with incomplete follow-up gained nothing at all. Even under that deliberately pessimistic assumption the lower limit of the confidence interval remains above the 8-point minimal detectable change for total SCIM III [19], although this observation describes the cohort trajectory and carries no implication of treatment effect in the absence of a comparator group.

A comparator group was sought at revision, as requested. The institutional database was interrogated for patients with traumatic cervical SCI treated with intensive rehabilitation during the same period without EES implantation, with the intention of assembling a group matched to the implanted cohort on age, baseline AIS grade, and time since injury. Serial SCIM III assessments at baseline and at 3, 6, 9, and 12 months—the outcome required for a like-for-like comparison—had, however, been recorded in only a minority of the patients identified, because SCIM III was collected systematically as part of the EES pathway but not as part of routine non-EES rehabilitation at this centre during the same period. A matched comparison of adequate size and equivalent outcome ascertainment could therefore not be assembled from existing records without introducing a further, and larger, source of bias, namely differential outcome measurement between groups. Rather than present an underpowered and differentially ascertained comparison as though it were a control group, we specify the comparison that is required and the exact form it should take. Table 2 sets out that matched-cohort analysis prospectively: 26 rehabilitation-only patients matched 1:1 to implanted patients on age (±5 years), baseline AIS grade, neurological level (C1–C4 versus C5–C8), and time since injury (±12 months), with propensity-score matching on these covariates and standardised mean differences reported for balance assessment [23], SCIM III collected at identical intervals by an assessor blinded to group, and change in total SCIM III from baseline to 12 months as the primary endpoint. This analysis is the immediate next step in our programme and is presented here as a specified protocol rather than as a result, because presenting an incomplete or non-equivalent comparison would misrepresent the strength of the evidence actually available.

## 4. Discussion

This study describes the safety profile and 12-month functional course of one of the larger single-centre EES cohorts assembled specifically in traumatic cervical SCI. The primary observations were that thoracolumbar EES implantation and a structured, individualised rehabilitation-and-stimulation pathway were feasible, that the observed device- and procedure-related adverse event rates were within the range reported for implanted spinal cord stimulation systems in other surgical series, and that total SCIM III scores increased over 12 months in the participants with complete follow-up. These indirect safety comparisons should be interpreted cautiously because the published series involve different populations and clinical indications. We deliberately do not frame the study as prognostic. The strong correlation between 3- and 12-month SCIM III scores is expected because both assessments use the same instrument in the same participants and are strongly related to baseline status; it therefore reflects stable longitudinal tracking rather than a novel predictive signal.

Domain-specific analysis demonstrated that functional recovery was not limited to mobility. Improvements were also observed in self-care and respiration and sphincter management, suggesting that the combined EES–rehabilitation program may influence multiple clinically relevant dimensions of independence. In patients with traumatic cervical SCI, gains in self-care and autonomic function may translate into meaningful reductions in caregiver burden and improvements in quality of life. Nevertheless, given the observational design and concurrent rehabilitation, these findings should be interpreted as descriptive rather than as evidence of a causal treatment effect. The graphical representation of both domain-specific trajectories (Figure 3) and individual patient responses (Figure 4) further illustrates the heterogeneity of recovery observed in this cohort.

To our knowledge, this is one of the largest single-cohort series of EES in traumatic cervical SCI, a subgroup previously described almost exclusively through case reports and small-N studies. The intended contribution is therefore primarily descriptive: it adds real-world safety and longitudinal functional data in an under-reported population and helps define the feasibility and adverse-event profile that an adequately powered, controlled trial would need to build on. We do not claim prognostic utility for the 3-month score.

On the methodological question of coupling: even when absolute rather than change scores are correlated, the strong dependence of both the 3- and 12-month scores on baseline (r ≈ 0.80–0.81) means the 3-to-12-month correlation is carried by stable between-patient differences already present at baseline. Reporting absolute scores does not remove this coupling; it re-expresses it. We therefore present the correlation descriptively only and did not fit an adjusted regression, which would be non-identifiable at this sample size. This is consistent with long-standing cautions about mathematical coupling and about over-parameterised models in small samples [27], and with the derivation-and-validation standards used in SCI prognostic work [28,29].

The clinical context remains important. Cervical SCI is associated with especially high disability because injury at this level affects not only locomotion but also upper-extremity use, trunk stability, transfers, sphincter management, and often respiration. SCIM III was therefore an appropriate outcome measure, as it captures domains directly relevant to systemic functions and independence [5,6,7]. The observed longitudinal increase in SCIM III is broadly consistent with the wider neuromodulation literature, in which EES combined with rehabilitation has been associated with improvements in voluntary movement, standing, stepping, trunk control, and overground walking in selected chronic SCI populations [10,11,12,13,14,15,16]. Consistent with patient-reported priorities, regaining upper-extremity function, self-care, and bowel and bladder control ranks among the most highly valued outcomes for individuals with cervical-level injuries [30], reinforcing the clinical relevance of the SCIM-based outcomes examined here.

Our cohort reflects real-world practice rather than a tightly protocolised experimental intervention, and programming had to be fundamentally patient-specific. There was no single amplitude, frequency, or pulse-width combination that was predictably effective across all patients; instead, stimulation settings were adjusted according to the patient’s functional priorities, visible and palpable muscle recruitment, movement quality, spasticity pattern, and autonomic responses. While this individualised approach reflects real-world care, it also introduces treatment heterogeneity that limits standard inferential interpretation, mirroring broader inconsistencies in electrode configuration, stimulation parameters, and rehabilitation protocols noted across the wider EES literature [17]. It is also possible that the heterogeneous responses observed across individuals reflect differences in the adequacy of stimulation programming rather than fixed biological limits on recovery; a standardised, prospectively documented programming protocol would help disentangle these explanations.

The concurrent use of robot-assisted gait and upper-extremity training in this cohort is supported by the broader rehabilitation robotics literature, which indicates that robot-assisted gait training can improve gait speed, walking distance, and lower-extremity strength in individuals with SCI, although evidence for its independent effect on balance, spasticity, and quality of life remains limited [31]. Critically, because this intensive robotic and conventional rehabilitation was delivered concurrently with stimulation in non-naive patients and without a rehabilitation-only comparator, the observed SCIM III gains cannot be partitioned between stimulation and training; any of the functional change reported here could, in principle, be attributable to rehabilitation or to continued spontaneous recovery rather than to EES. This descriptive study was not designed to isolate a single treatment component, but rather to document the safety and functional course of EES delivered within an integrated, individualised rehabilitation pathway as it is used in real-world practice. This limitation is not unique to the present cohort: landmark EES studies have similarly combined stimulation with structured, often intensive rehabilitation programs [12,14,15].

Interpreting the magnitude of the observed gains against natural history requires caution. In chronic (more than 12 months), motor-complete traumatic cervical SCI, spontaneous improvement in independence under conventional rehabilitation alone is generally small, because most measurable neurological and functional recovery occurs within the first year after injury, and clinically meaningful SCIM self-care gains are largely confined to individuals who recover two or more motor levels [32,33]. The present cohort was predominantly chronic (median injury-to-implant interval 57 months), although a minority were implanted in the late subacute period, as early as 10 months after injury, when some spontaneous recovery may still occur. The statement in the previous version that the observed changes appeared larger than would be expected from natural history alone has been withdrawn, and the extended justification that accompanied it has been shortened accordingly, as the reviewer suggests. No matched historical or concurrent comparator was available; published natural-history estimates derive from cohorts differing in case mix, era, rehabilitation intensity, and outcome ascertainment; and an indirect comparison of that kind cannot establish that the observed change exceeds what conventional rehabilitation alone would have produced in these particular patients. The trajectory is therefore reported without benchmarking; the matched comparison specified in Table 2 is what would be required to benchmark it, and this caution applies most strongly to the subacute minority, in whom continuing spontaneous recovery remains a plausible explanation.

Regulatory and ethical considerations warrant explicit comment. EES for motor and autonomic restoration is investigational, and the devices used are cleared for chronic pain and were applied off-label here with an explicit therapeutic goal; implanting an active device for an experimental indication is an interventional act, and we do not characterise the underlying clinical activity as risk-free or exempt from oversight. The cohort included a young adult (Patient 27) with a complete C2–C3 injury and a SCIM III score of 0 at every time point; an experimental implant of this kind requires clear justification of indication and a realistic account of expected benefit, and the absence of measurable change in this patient is reported transparently rather than omitted. These considerations reinforce that EES in this setting should proceed within carefully governed, prospectively designed frameworks.

Beyond locomotor and upper-extremity domains, autonomic recovery represents a further dimension of clinical importance in cervical SCI. Although the SCIM III instrument captures sphincter management as one of its subdomains, the present analysis did not separately report bladder, bowel, or cardiovascular outcomes. Prior work has shown that epidural stimulation of the lumbosacral spinal cord can improve voiding function and modulate bowel activity after chronic SCI without adversely affecting cardiovascular stability [34]. Because autonomic-oriented programming was incorporated for selected patients, future analyses using dedicated urodynamic and bowel-function instruments would help clarify whether autonomic gains parallel the SCIM-based functional improvements reported here.

The safety profile observed in this cohort included a 6.3% (2/32) rate of persistent sterile inflammatory reactions and an overall explantation rate of 3.1% (1/32). These adverse event rates were within the range reported for implanted spinal cord stimulation systems in other surgical series, although direct comparisons should be interpreted cautiously because of differences in patient populations, clinical indications, and treatment pathways. Multicentre registry data on spinal cord stimulation for chronic pain have reported infection rates of approximately 2.4–3.8% and annual explantation rates of 6–9%, with hardware-related infection and loss of therapeutic benefit among the most common reasons for device removal [35]. Viewed in this context, the complication profile observed in the present cohort does not appear unexpected, although the relatively small sample size limits the precision of these estimates. The single first-ever seizure accompanied by EEG abnormalities during follow-up after EES implantation is noteworthy. Although a causal relationship cannot be established from this observational study, the occurrence of a new-onset seizure in a patient without a previous history of epilepsy supports continued mechanistic investigation and prospective neurophysiological monitoring in future EES studies in traumatic cervical SCI.

The limitations of this study are more consequential than a conventional list conveys, and they are therefore set out individually below. To remove the duplication noted at review, each limitation is stated once, at the point at which it bears on interpretation, and is cross-referenced rather than restated in the Methods and the Results. Three constraints that require no separate development are recorded here. The cohort was drawn from a single institutional database, which limits generalisability. The dataset was not designed for a formal adverse-event analysis, although the descriptive safety observations are consistent with routine clinical follow-up and are reported under the definitions given in Section 2.8. And repeated comparisons across follow-up time points were interpreted descriptively, with Holm correction where a family of comparisons was reported, rather than as confirmatory hypothesis tests. The eight limitations that do bear individually on interpretation follow.

First, the baseline distribution is extremely narrow. Thirty-one of 32 patients had a baseline AIS grade of A and one patient had AIS B, and no patient with a baseline grade of C entered the cohort even though the eligibility criterion permitted it. With a single AIS B patient, any analysis involving baseline severity is in substance an analysis of that one individual; and because that same patient showed the largest improvement in the cohort (total SCIM III 30 to 82, with conversion to AIS D), a single observation exerts disproportionate influence on the converter versus non-converter contrast, on the upper end of the 3-to-12-month correlation, and on the cohort mean change. The cohort should accordingly be read as a description of motor-complete (AIS A) traumatic cervical SCI, and nothing in these data supports extrapolation to incomplete injuries.

Second, 28 of the 32 patients (87.5%) were male. Although this reflects the epidemiology of traumatic SCI, a sample of four women provides almost no information about outcomes in women, in whom anthropometric, hormonal, bladder-management, and caregiving differences could plausibly influence both the response to stimulation and the scoring of SCIM III items. Generalisability to women is correspondingly weak, and future studies should be powered to report outcomes disaggregated by sex rather than treating sex as a nuisance covariate. For the same reason, no sex-disaggregated outcome analysis is presented in Section 3; the basis for that decision is set out there.

Third, the injury-to-implant interval spans 10 to 339 months, a more than thirty-fold range, and these are not clinically equivalent patients. A patient implanted 10 months after injury remains within the period in which spontaneous neurological and functional recovery is still documented, whereas after 339 months spontaneous recovery is essentially not expected. Reporting a single mean of 64.3 ± 66.7 months conceals that heterogeneity, and applying the same protocol across the whole range makes chronicity an uncontrolled confounder of the observed trajectory. The analysis should be stratified by chronicity (<12, 12–60, and >60 months), which the present sample size cannot support and which is therefore specified as a prespecified subgroup analysis in Table 2.

Fourth, one patient had a multilevel injury with cervical (C4) and additional thoracolumbar involvement. This patient met the stated inclusion criterion, but a multilevel lesion is not equivalent to an isolated cervical lesion with respect either to the segmental target of thoracolumbar stimulation or to the interpretation of SCIM III mobility items, and its inclusion adds heterogeneity to an already skewed baseline. With a single such patient in 32 the influence on the cohort mean is bounded but not negligible, and exclusion of multilevel injuries is a prespecified eligibility criterion of the comparative study proposed in Table 2.

Fifth, and most consequentially, there was no control group of any kind. Rehabilitation was intensive, prolonged, and delivered concurrently to patients who were not rehabilitation-naive, and continuing spontaneous recovery cannot be excluded in the subacute minority. No analysis of these data can separate the contribution of stimulation from that of rehabilitation or of elapsed time. We agree with the reviewers that a propensity-matched comparison against contemporaneous rehabilitation-only patients is the minimum acceptable design for any future effectiveness claim, and that a randomised or stepped-wedge design would be preferable; Table 2 specifies such a comparison prospectively.

Sixth, the intervention as delivered is not reproducible. Stimulation parameters were individualised without a written prospective protocol, and although the parameter space and the titration rule that was applied are now reported in structured form (Section 2.5 and Table 1), another centre could not reconstruct the exposure received by any individual patient in this cohort. This is a limitation of the exposure itself and not merely of its reporting, and it means that the present results cannot serve as a benchmark against which another centre’s results could legitimately be compared.

Seventh, outcome assessment was unblinded, was performed by clinicians belonging to the treating team, and was carried out with the device active. Observer and expectation bias cannot be excluded, and the reported scores describe function with stimulation in use rather than a stimulation-attributable increment.

Eighth, longitudinal analysis was restricted to 26 of 32 patients. A conservative baseline-observation-carried-forward analysis covering all 32 implanted patients is now reported alongside the complete-case result, and the 6 patients without complete follow-up are accounted for in Section 3. Nevertheless, 6 incomplete follow-ups in a cohort of 32 is an attrition rate of 18.8%, the post-baseline trajectories of those 6 patients are unknown, and no statistical technique can recover observations that were never made. The practical implication is that the complete-case estimate of +17.62 points should be read as the most favourable of the available estimates and the baseline-observation-carried-forward estimate of +14.32 points as its lower bound, with the true cohort value lying between them.

A further limitation is that rehabilitation dose and adherence were estimated from therapist logs and patient reports rather than objectively measured. Future prospective studies should use wearable sensors or electronic diaries to quantify actual therapy time.

Given the descriptive objective of this study, the relatively small sample size, and the limited number of repeated observations, longitudinal changes were summarised using paired within-subject comparisons rather than linear mixed-effects modelling. The present analyses were intended to describe functional trajectories rather than to estimate population-level longitudinal effects or to develop predictive models.

The broader question of whether early status can inform later functional prognosis has previously been addressed through formal clinical prediction rules in other SCI contexts. A multicentre European cohort study derived and validated a prediction rule for ambulation outcome after traumatic SCI using age and early neurological examination findings [28], and a more recent cervical-specific model combining age, sex, and early sensorimotor scores predicts upper-limb functional dependency at one year [29]. These precedents illustrate both the feasibility and the methodological rigour required to move from a descriptive observation toward a validated clinical prediction tool. Future studies of EES in traumatic cervical SCI would benefit from a similarly structured derivation-and-validation framework, ideally in multicentre cohorts with prespecified statistical analysis plans and STROBE-compliant reporting [22].

Despite these constraints, the cohort remains clinically informative. Traumatic cervical SCI is a high-burden subgroup in which even partial gains may have important real-life consequences, and longitudinal safety and functional datasets in such patients remain relatively limited. The present findings support continued investigation of EES within individualised, rehabilitation-integrated care pathways under prospective, controlled, and adequately governed designs.

Our broader clinical programme now includes individualised cervical, thoracolumbar, and combined cervical–thoracolumbar epidural stimulation strategies for patients with traumatic cervical SCI. The present study, however, reports exclusively the thoracolumbar stimulation cohort in order to maintain a homogeneous stimulation strategy; patients treated with cervical or combined cervical–thoracolumbar stimulation were intentionally excluded from the present analysis and will be reported separately after completion of prospective follow-up. Throughout this programme, functional changes have been prospectively documented using serial SCIM III assessments and standardised video recordings.

Thoracolumbar epidural stimulation in this cohort was frequently accompanied by improvements in upper-extremity function despite the absence of direct cervical stimulation. Clinically, we observed activation of the triceps with stable elbow extension and locking in a number of patients, sometimes together with gross grasp and, less often, pinch and precision grip. We deliberately refrain from proposing a mechanism for this observation. No electromyographic, evoked-potential, transcranial magnetic stimulation, or imaging data were acquired in this cohort, and without such measurements the functional recruitment of preserved supraspinal and propriospinal networks is only one of several possible explanations. Others include non-specific effects of the intensive concurrent upper-limb rehabilitation and robotic training delivered to every patient, improved trunk and pelvic stability permitting more effective use of the arms, reduction in spasticity, changes in posture and seating, and observer expectation in an unblinded assessment. The observation is therefore reported as a clinical description that requires explanation rather than as evidence of a propriospinal mechanism, and the earlier mechanistic wording has been removed. Dedicated neurophysiological measurement within a prospectively designed, blinded study would be required before any mechanistic claim of this kind could responsibly be made.

## 5. From Description to Practice: A Transparent Protocol Framework

The reviewers ask, rightly, that this report move beyond an implicit “it appears to work” towards an explicit account of how it should guide practice. Our position is as follows. This study does not establish that EES is effective in traumatic cervical SCI, and it should not be cited as evidence that it is. What it does provide is precisely the information that a properly controlled study needs and that the existing literature—largely single cases and small series—does not yet supply: a device- and procedure-related adverse-event profile in 32 consecutive cervical implantations with continuous safety surveillance extending beyond 12 months; a quantified rehabilitation dose against which future protocols can be calibrated; a structured description of the stimulation parameter space and of the clinical endpoints used to titrate it; and realistic estimates of attrition, assessment burden, and outcome variability with which a future trial can be powered.

On that basis we make three specific recommendations for practice today. First, EES for restorative indications in traumatic cervical SCI should be performed only within a prospectively registered, ethics-approved protocol with independent safety monitoring, and not as individually justified off-label care; the present cohort is offered as the safety and feasibility evidence on which such a protocol can be built, not as a warrant for continued uncontrolled use. Second, wherever EES is performed, the minimum reporting set specified in Table 1 should be recorded prospectively for every patient, so that the exposure is characterised well enough for independent replication. Third, functional outcome should be assessed by an assessor blinded to group and, where feasible, in both stimulation-on and stimulation-off conditions, with rehabilitation dose and adherence quantified objectively, so that the contribution of stimulation can be separated from that of the rehabilitation delivered alongside it. Table 1 sets out this framework in full, and it is the practical contribution that we intend this report to make.

## 6. Conclusions

This is a descriptive safety-and-feasibility study and not an efficacy study; the findings below describe what was observed and do not constitute evidence that EES is effective. In this descriptive cohort of patients with traumatic cervical SCI, thoracolumbar EES combined with structured, individualised rehabilitation was feasible, and total SCIM III scores increased over 12 months among the participants with complete follow-up. The observed adverse event rates were within the range reported for implanted spinal cord stimulation systems in other surgical series; however, these indirect comparisons should be interpreted cautiously because they involve different patient populations and clinical indications. Because there was no comparator group and rehabilitation was delivered concurrently and intensively, the observed functional changes cannot be attributed to stimulation alone. The close correlation between 3- and 12-month SCIM III scores reflects stable tracking of a repeated measure rather than established prognostic value. These hypothesis-generating findings should be examined in prospectively designed, adequately powered controlled studies with prespecified statistical analysis plans and clear regulatory and ethical governance. The practical value of this report therefore lies not in the magnitude of the gains observed but in the transparency of the safety, dose, programming, and attrition data it provides, and in the explicit protocol framework (Table 6) and matched-comparator specification (Table 5) through which those data can be converted into clinically actionable evidence.

## Figures and Tables

**Table 1:** Proposed transparent protocol and minimum reporting framework for the controlled evaluation of epidural electrical stimulation in traumatic cervical spinal cord injury.

Domain	Element to Be Prespecified and Reported	Specification Recommended on the Basis of the Present Cohort
Eligibility	Injury type, level, AIS grade, chronicity window	Traumatic cervical SCI; AIS grade window stated and enforced; chronicity stratified (<12, 12–60, >60 months); multilevel injuries excluded or analysed separately
Comparator	Group allocation	Contemporaneous rehabilitation-only comparator, 1:1 propensity-matched on age, AIS grade, level, and chronicity; randomised or stepped-wedge allocation preferred
Lead target	Anatomical level, lead type, contact map	Paddle lead, T11–L1; rostrocaudal position and contact numbering reported per patient with intraoperative fluoroscopy
Programming—screening	Fixed initial screening sequence	Predefined contact configurations tested in fixed order; surface EMG motor-threshold determination for quadriceps, hamstrings, gluteals, and tibialis anterior
Programming—parameters	Frequency, pulse width, amplitude, duty cycle per program	Motor facilitation 20–40 Hz; inhibitory/antispasticity 900–1200 Hz; pulse width 600–1000 µs; amplitude 1–25.5 mA titrated in 0.1–0.5 mA steps; each program reported separately by functional target
Programming—titration rule	Endpoint used to set amplitude	Lowest amplitude producing the reproducible functional endpoint (e.g., knee extension sufficient for supported stance); re-checked monthly; parameter set locked for a defined block before any change
Programming—reporting	Stimulation parameters	Per-patient parameter data are available from the corresponding author upon reasonable request.
Rehabilitation dose	Prescribed and delivered therapy hours	Prescribed dose stated (present cohort ≈ 84 supervised + ≈1003 home = ≈1087 h over 12 months); delivered dose measured, not assumed
Adherence and fidelity	Measurement method	Wearable sensors or electronic diaries; therapist-verified fidelity checklist at fixed intervals
Outcome assessment	Instrument, assessor, condition, timing	SCIM III and ISNCSCI; assessor blinded to group and to stimulation condition; assessed both stimulation-on and stimulation-off; baseline and 3, 6, 9, and 12 months
Interpretation thresholds	MCID and MDC	Total SCIM III: MCID ≈ 4 points, MDC ≈ 8 points, substantial improvement ≈ 10 points [19]
Statistical plan	Primary model and missing-data strategy	Prespecified linear mixed-effects model on all implanted patients; likelihood-based analysis under missing-at-random with baseline-observation-carried-forward sensitivity; exact adjusted *p* values reported
Safety	Definitions, causality grading, surveillance window	ISO 14155:2020 definitions [20] with WHO–UMC causality grading [21]; safety surveillance of at least 24 months, reported separately from the functional-assessment window
Safety	Independence of causality adjudication	Causality graded independently by two assessors with no role in implantation or stimulation programming and, preferably, external to the treating centre; adjudication procedure and disagreement-resolution rule prespecified.
Governance	Regulatory and ethical framework	Prospective registration; ethics-approved protocol; independent data and safety monitoring board; explicit off-label consent; declared presence or absence of manufacturer relationships
Reporting	Subgroup and sex-disaggregated outcomes	Outcomes reported separately for women and men and by chronicity stratum; minimum enrolment of women prespecified; statistical software, package names, and version numbers reported, with the analysis script deposited alongside the protocol.

AIS, American Spinal Injury Association Impairment Scale; EMG, electromyography; ISNCSCI, International Standards for Neurological Classification of Spinal Cord Injury; MCID, minimal clinically important difference; MDC, minimal detectable change; SCI, spinal cord injury; SCIM, Spinal Cord Independence Measure.

**Table 2:** Prespecified matched-comparator (rehabilitation-only) analysis.

Element	Specification
Comparator source	Institutional rehabilitation database, same centre, same calendar period; traumatic cervical SCI treated without EES
Sample size	26 comparator patients, matched 1:1 to the implanted cohort
Matching variables	Age (±5 years); baseline AIS grade; neurological level (C1–C4 vs. C5–C8); time since injury (±12 months)
Matching method	Propensity-score matching (nearest neighbour, calliper 0.2 SD of the logit), with exact matching on baseline AIS grade
Balance assessment	Standardised mean differences for every covariate before and after matching; |SMD| < 0.10 accepted as balanced [23]
Rehabilitation exposure	Prescribed and delivered therapy hours recorded in both groups so that dose, not group label, is the compared exposure
Outcome measure	Total and domain SCIM III at baseline and 3, 6, 9, and 12 months; ISNCSCI/AIS at baseline, 3, and 12 months
Outcome assessment	Assessor blinded to group allocation and, in the implanted group, to stimulation condition
Primary endpoint	Between-group difference in change in total SCIM III from baseline to 12 months
Primary analysis	Linear mixed-effects model with group × time interaction on all matched patients; missing data handled under a missing-at-random assumption
Sensitivity analyses	Baseline-observation-carried-forward imputation; stratification by chronicity (<12, 12–60, >60 months); exclusion of multilevel injuries
Statistical software and reproducibility	Matching, balance assessment, and the primary model performed in R version 4.4.1 or later: MatchIt for nearest-neighbour matching on the logit of the propensity score (calliper 0.2 SD, exact matching on baseline AIS grade) [24]; cobalt for standardised mean differences before and after matching; lme4 and lmerTest for the linear mixed-effects model. Propensity-score model, matching call, and analysis script deposited with the protocol. Descriptive analyses of the present cohort were performed in IBM SPSS Statistics version 29.0.
Prespecified subgroup reporting	Outcomes reported disaggregated by sex, with a minimum enrolment of women prespecified at the design stage, and by chronicity stratum. The present cohort (4 women of 32) cannot support such reporting.
Safety adjudication	Adverse-event causality graded independently, under ISO 14155:2020 definitions and WHO–UMC categories, by two assessors external to the implanting and programming team, with a third adjudicator for unresolved disagreement.
Status	Specified and approved; not yet executed. No comparative effectiveness claim is made in the present report.

AIS, American Spinal Injury Association Impairment Scale; EES, epidural electrical stimulation; ISNCSCI, International Standards for Neurological Classification of Spinal Cord Injury; SCI, spinal cord injury; SCIM, Spinal Cord Independence Measure; SMD, standardised mean difference.

**Table 3:** Baseline characteristics of the traumatic cervical SCI cohort (*n* = 32).

Variable	Value
Patients, *n*	32
Male sex, *n* (%)	28 (87.5)
Age, years, mean ± SD	33.8 ± 10.3
Age, years, median (range)	30 (21–53)
Baseline AIS grade A/B, *n*	31/1
Injury-to-implant interval, months, mean ± SD	64.3 ± 66.7
Injury-to-implant interval, months, median (range)	57 (10–339)

AIS, American Spinal Injury Association Impairment Scale; SCI, spinal cord injury; SD, standard deviation.

**Table 4:** Longitudinal total SCIM III scores in participants with complete 12-month follow-up (*n* = 26).

Assessment Time	Mean ± SD	Median (IQR)	Mean Change from Baseline	*p* Value *
Baseline	14.81 ± 8.92	12.0 (12.0–16.25)	–	–
3 months	21.77 ± 14.60	17.0 (14.0–27.50)	+6.96	<0.001
6 months	25.12 ± 15.90	21.0 (15.25–34.00)	+10.31	<0.001
9 months	28.23 ± 17.68	23.5 (17.25–37.50)	+13.42	<0.001
12 months	32.42 ± 19.45	28.0 (18.75–42.75)	+17.62	<0.001

Values are presented as mean ± standard deviation (SD) and median (interquartile range, IQR). * Wilcoxon signed-rank test versus baseline, Holm-adjusted. The Holm-adjusted *p* value was below 0.001 at every time point. With 26 paired observations the smallest two-sided p attainable by the exact Wilcoxon signed-rank test is approximately 3 × 10^−8^, so the adjusted values are reported against the 0.001 threshold rather than as point estimates, which at this magnitude would convey spurious precision; adjusted values of 0.001 or greater are reported to three decimal places wherever they occur. Longitudinal analyses include only the 26 participants with complete follow-up through 12 months (complete-case analysis). SCIM, Spinal Cord Independence Measure.

**Table 5:** Longitudinal SCIM III domain scores in participants with complete 12-month follow-up (*n* = 26).

Assessment Time	Self-Care	Respiration & Sphincter Management	Mobility	Total SCIM III
Baseline	1.92 ± 2.74	11.04 ± 4.85	1.85 ± 2.39	14.81 ± 8.92
3 months	3.62 ± 4.41	14.54 ± 7.21	3.62 ± 3.95	21.77 ± 14.60
6 months	4.12 ± 4.57	16.15 ± 7.56	4.85 ± 4.66	25.12 ± 15.90
9 months	5.15 ± 5.09	17.19 ± 8.35	5.88 ± 5.28	28.23 ± 17.68
12 months	6.08 ± 5.31	19.27 ± 9.20	7.08 ± 6.04	32.42 ± 19.45

Values are presented as mean ± SD. Longitudinal analyses include only the 26 participants with complete follow-up. SCIM, Spinal Cord Independence Measure; SD, standard deviation.

**Table 6:** Individual change in total SCIM III score from baseline to 12 months in participants with complete follow-up (*n* = 26).

Outcome	Value
Baseline total SCIM III, mean ± SD	14.81 ± 8.92
12-month total SCIM III, mean ± SD	32.42 ± 19.45
Mean change, baseline to 12 months	+17.62 ± 13.10
95% CI of mean change	12.33 to 22.91
Median change (IQR)	13.5 (7.5–21.0)
Minimum change	0
Maximum change	+52
Participants with ≥10-point improvement, *n* (%)	18/26 (69.2)
Participants with <10-point improvement, *n* (%)	8/26 (30.8)

CI, confidence interval; IQR, interquartile range; SCIM, Spinal Cord Independence Measure; SD, standard deviation.

## Data Availability

The data supporting the findings of this study are not publicly available due to privacy and ethical restrictions. The datasets generated and/or analyzed during the current study are available from the corresponding authors upon reasonable request.

## Abbreviations

AIS	American Spinal Injury Association Impairment Scale
EES	Epidural Electrical Stimulation
EEG	Electroencephalography
SCI	Spinal Cord Injury
SCIM III	Spinal Cord Independence Measure III
AE	Adverse Event (ISO 14155:2020)
BOCF	Baseline Observation Carried Forward
CI	Confidence Interval
EMG	Electromyography
ISNCSCI	International Standards for Neurological Classification of Spinal Cord Injury
LMM	Linear Mixed-effects Model
MCID	Minimal Clinically Important Difference
MDC	Minimal Detectable Change
SMD	Standardised Mean Difference
STROBE	Strengthening the Reporting of Observational Studies in Epidemiology
WHO-UMC	World Health Organization—Uppsala Monitoring Centre causality assessment system
